# Does dietary diversity predict the nutritional status of adolescents in Jimma Zone, Southwest Ethiopia?

**DOI:** 10.1186/s13104-019-4437-3

**Published:** 2019-07-15

**Authors:** Birhanu Jikamo, Mekonen Samuel

**Affiliations:** 1 College of Medicine and Health Sciences, School of Public Health, Hawassa University, Hawassa, Ethiopia; 2 Hossaena Town Communicable Disease Control Clinical Officer, Hosanna, Ethiopia

**Keywords:** Nutritional status, Dietary diversity, Adolescent, Ethiopia

## Abstract

**Objective:**

This study aimed to assess the association between dietary diversity and nutritional status of adolescents in Jimma Zone, Southwest Ethiopia. A secondary data was used to assess the association between dietary diversity and nutritional status of adolescents in Jimma Zone, Southwest Ethiopia. Adolescents in the age of 13–17 years old included in the analysis. The data cleaned, coded and analyzed using Stata version 14.

**Results:**

Over all prevalence of stunting and thinness were 26.1% and 25.3%. Stunting was higher among female adolescents (23.8%) than male (21.9%), while thinness was higher among male (27.5%) than female (25.3%) adolescents. In multivariate binary logistic regression model, being female adolescents were 98% (AOR = 1.98; 95% CI 1.6, 2.4] higher odd of stunted compared with those male adolescents, households had food insecurity were 67% [AOR = 1.67; 95% CI 0.6, 0.9] more likely to be associated with stunted than with those households which had secure foods. Adolescents who had high workload were 2.6 times [AOR = 2.6; 95% CI 1.2, 3.1] more likely to be associated with thinness compared with those adolescents who didn’t had high workload.

## Introduction

In, 2017 international report indicated that 1.8 billion estimated adolescents were live, from these 90% of them were from Low and Middle Income Countries (LMICs) [1]. Similarly, in Ethiopia in 2010 report showed that from the total population more than quarters (25.9%) of them were adolescents [2]. For the reason that, adolescents should provide special concentration not only their health and nutrition requirements but also they are our hope of problem-solvers, doctors, politicians and universal leaders [3].

In, 2017 South Asia report found that magnitude of moderate and severe underweight is highest compared with other countries; one in 5 girls aged 5–19 years and nearly 1/3rd of their male peers were underweight [4]. Adolescents lowest body mass index (BMIs) were registered in Ethiopia, Niger, Senegal, India, Bangladesh, Myanmar, and Cambodia [4].

Adolescents vulnerable to overweight and poor metabolic activities were associated with the happening of Non-Communicable Disease (NCD) and associated mortality later in life [5, 6]. In addition to this, global problem of moderate or severe proportion of underweight were higher than that of overweight and obesity [4, 5]. The adolescent nutritional problem is mainly public health concern in Sub-Saharan Africa where the percentage of underweight is much higher than that of overweight and obesity [4, 5].

Stunting is one of main public health crisis among adolescent girls because of adolescent pregnancy is associated adverse-birth outcome and sexual transmitted diseases were very common in international societies [7, 8]. Furthermore, adolescent pregnancy not only affects growth for numerous girls in the region of the world, it is also risk factors for fetal growth restriction, premature birth and early neonatal deaths [7, 8]. As the result of these, giving attention for adolescent nutrition and health is important to improve maternal and child health [7, 8].

For a number of years, the healths of adolescents and nutrition have not been given a major public health concern and there were no sufficient published literatures [8, 9]. Many of the global societies considers they were believed to be lesser susceptible to disease and suffer from fewer life-threatening conditions than those children and elderly people [8, 9]. Besides, adolescent nutrition receives very little attention and difficult to quantify accurately in this age group due to rapid change in growth and development and lack of consensus over which definition to use [8, 9].

Ethiopia, as the LMICs, nutritional problem is one of the major public health concerns for all phases of human life [9]. Moreover, in Ethiopia under nutrition is a main public-health catastrophe due to most people addicted non-diversified diet [10]. But, there is little evidence about the nutritional status of adolescents in Ethiopia [11, 12]. Furthermore, adolescent nutrition is distress from lack of documented evidences, low policymaker interest and little attention of program experience in the study setting [11, 12].

Responsible bodies will give consideration for adolescent nutrition which is potential to improve economic productivity of countries, good pregnancy outcomes, and minimize adverse-chronic disease outcomes [10, 12]. Moreover, in Africa specifically in Ethiopia and Tanzania higher magnitude of under nutrition among adolescents have been registered (27%) [10], and (21%) [13]. Consequently, exceptional concern is needed to adolescent dietary intake and practice, which is important contributor to get better adolescent health and productivity [10, 12].

Adolescents are frequently considered as healthy and strong but, previous literature indicated that numerous adolescent were underweight and stunted in height [11, 12]. In spite of this, previous literature in Ethiopia showed that still gave more consideration on vulnerable people dietary intake such as: infant, pregnant and lactating women but, limited evidences for adolescents [10, 11]. Understanding the association of determinants with adolescent’s dietary diversity in take, this is important to reduce under-nutrition among adolescents and gives specific intervention. Dietary diversity and nutritional status of adolescents in Ethiopia is significant problem. The findings from this study could be used to assess the association between dietary diversity and nutritional status of adolescents in Jimma Zone, Southwest Ethiopia.

## Main text

### Methods

#### Study design, setting and population

We analyzed secondary data collected from a community-based cross-sectional longitudinal survey conducted in selected woredas in Jimma zone, Southwest Ethiopia. The Jimma Longitudinal Family Survey on Youth (JLFSY) was conducted in 2005–2006. Adolescents in the age group of 13–17 years old included in the analysis.

#### Sample size and sampling procedure

Two-stage sampling technique used to select sample of adolescents. At the first stage, households were randomly selected from selected kebele based on proportional allocation of adolescents. In the second stage, one adolescent (a boy or a girl) was randomly selected from each household. A total of 2084 adolescents used for this analysis, of 1025 female and 1059 male.

#### Data collection tools and procedure

The data for prevalence of stunting and thinness and associated factors was taken from baseline survey of adolescents in Jimma zone. The variables reviewed were relevant for this research question were collected from the SPSS filled data.

Questionnaires translated into Amharic and Oromifa languages and checked for consistency by another person who speaks both Oromifa and English. The questionnaires focused on issues related to adolescents’ such as: practice of nutritional status, socio demographic, economic and environmental factors, food insecurity and anthropometric measurements of the adolescent included and used to address the research question.

Anthropometric measurements measured by trained health professionals by using mid upper arm circumference, weight and height of the study participants.

A Dietary Diversity Score (DDS) constructed by counting the intake of the food groups over a period of 1 week based on the definition that it is the sum of food groups consumed over the reference period. For example, an adolescent who consumed one item from each of the food groups at least once during the week would have the maximum DDS. The DDS converted into tertiles and the highest tertile used to define ‘‘high’’ dietary diversity score, “medium” score and while the two lower tertiles combined labeled ‘‘low’’ dietary diversity score.

We maintained quality of the data checked its completeness, cleaned the missing values by running frequencies and some of the variables re-coded into the same variable. Dietary diversity assessed using a food frequency questionnaire containing food items that were commonly consumed in the study setting.

#### Data processing and analysis

The data analyzed using Stata version 14. Frequency, percentage and descriptive summaries used to explain the amount of study participants in the analysis. Detection of specific factors associated with nutritional status among adolescents using binary logistic regression. Explanatory variables were significant in the binary logistic regression analysis with a cut-off point of P-value < 0.25 were candidate factors for the multiple binary logistic regression models [14].

In the multiple binary logistic regression models measure of strength of association were reported as odds ratio (OR) with 95% CIs, by controlling the effect of other factors. P-value of < 0.05 was indicated significant association between the nutritional status and the independent predictors.

Stepwise forward method of model building technique was used for each model development. Confounders and interaction effects were assessed by calculating relative changes on ß coefficients with a cutoff point 15% [15]. Multicolinearity effect was assessed with cut off point mean of variation inflation factor (VIF) less than five [16] and model robustness was also assessed using Hosmer and Lemeshow techniques [17].

Dietary diversity obtained by summing the number of food and food items consumed in each group separately [18–20]. The total score calculated and this ranged from 0 to 12. Tertiles of DDS used to classify the children into low (≤ 4), medium (5–8) and high (9–12) [18, 19].

Anthropometric measurements: three indicators assessed such as: weight-for-age, height-for-age and weight-for-height Z-scores for all the adolescents. The weight was measured using portable standing scale. The weight was recorded to the nearest 0.1 kg. It was calibrated against known weight regularly. During the procedure the subjects wore light clothes and bare foot. The level of stunting (height for age *z*-scores), which was an indicator of chronic malnutrition, and thinness (BMI for age *z*-scores), which was another indicator of malnutrition, were calculated using WHO Athro-Plus software [21]. Thus, those below − 2 standard deviations of the NCHS median reference for height-for-age and weight-for-height were defined as stunting [22].

### Results

#### Socio-demographic characteristics of the study participants

A total of 2084 participants were interviewed: 1059 (50.8%) were boys with a mean age of 14.78 years and a standard deviation of 1.34. More than one-fourth 793 (38.1%) of study participants were reside in rural communities (Table 1).

**Table 1:** Socio-demographic characteristics of study adolescents in Jimma Zone, Ethiopia, October, 2015 (N = 2084)

Variables	Category	Frequency (n = 2084)	Percent (%)
Residence	Urban	734	35.2
	Semi-urban	557	26.7
	Rural	793	38.1
Sex	Male	1059	50.8
	Female	1025	49.2
Mother’s educational status	No education	717	34.4
	Primary education	918	44.1
	Secondary education	317	15.2
	College and above	132	6.3
Father’s educational status	No education	603	28.9
	Primary education	655	32.5
	Secondary education	559	26.8
	College and above	267	12.8
Age in years mean (± SD)	14.78 (± 1.34)		
Household income mean (± SD)	105.77 (± 188.13)		
Household size mean (± SD)	8.49 (± 3.42)		
Highest-grade completed mean (± SD)	5.16 (± 2.66)		

In the multivariable analysis, residence, sex of adolescent, and households food insecurity had significantly associated with being stunted (Table 2).

**Table 2:** Bivariate and multivariable logistic regression analysis with stunting of Jimma zone, Ethiopia, (JLFSY2005-2006) (N = 2084)

Variables	Nutritional status by stunted							
	Category	Stunted		COR	95% (CI)	AOR	95% (CI)	P-value
		Yes	No					
Residence	Urban (ref)	147	408	1	1		1	
	Semi urban	203	529	0.9	(0.7, 1.2)^a^	0.93	(0.7, 1.2)	0.16
	Rural	194	477	0.33	(0.8, 1.3)^b^	1.83	(1.2, 3.1)**	0.001
Sex of adolescent	Male (ref)	214	759	1	1		1	
	Female	330	655	0.5	(0.5, 0.7)^b^	1.98	(1.6, 2.4)***	0.0001
Household food insecurity	Secure (ref)	198	560	1	1		1	
	Insecure	346	854	0.8	(0.7, 1.07)^a^	1.67	(0.6, 0.9)*	0.025
Adolescent food insecurity	Secure (ref)	45	160	1	1		1	
	Insecure	499	1254	0.7	(0.5, 1.05)^a^	0.65	(0.4, 0.9)	0.055
Dietary diversity	Low (ref)	203	578	1.3	1		1	
	Medium	92	241	0.9	(0.9, 1.4)^a^	1.3	(.99, 1.8)	0.067
	High	249	595	0.9	(0.8, 1.4)^a^	0.29	(1.06, 1.6)**	0.001
High work load of adolescents	No (ref)	116	315	1	1		1	
	Yes	428	1099	0.4	(0.9, 1.2)^b^	1.3	(0.9, 1.7)	0.065

* Significant results 1-reference category. Statistical significant (*p < 0.05), highly significant (** p ≤ 0.001) *** p ≤ 0.0001, * p ≤ 0.05^b^, p ≤ 0.25^a^

After adjusted binary logistic regression analysis, rural adolescents had 83% [AOR = 1.83; 95% CI 1.2, 3.1] higher odds of being stunted compared with those adolescents who resided in urban areas. Female adolescents had 98% (AOR = 1.98; 95% CI 1.6, 2.4] higher odds of being stunted compared with male adolescents. Households’ with food insecurity were 67% [AOR = 1.67; 95% CI 0.6, 0.9] more likely to be associated with stunting compared with those households which had secure foods (Table 2).

During multivariable analysis the following variables were associated with being thinness, residence, household’s food insecurity and high work load of adolescents (Table 3).

**Table 3:** Bivariate and multivariate logistic regression table for thinness of Jimma zone, Ethiopia (JLFSY2005-2006) (N = 2084)

Variables	Nutritional status by underweight (thinness)							
	Category	Thinness		COR	95% (CI)	AOR	95% (CI)	P-value
		Yes	No					
Resident	Urban (ref)	122	610		1		1	
	Semi urban	174	494	0.6	(1.6, 2.8)^b^	1.2	(1.2, 2.1)	0.056
	Rural	169	384	0.8	(1.3, 2.3)^b^	1.6	(1.3, 2.2)***	0.0001
Mother educational status	No education (ref	21	111		1		1	
	Primary education	72	263	3.7	(0.9, 4)^a^	0.9	(0.6, 1.2)	0.520
	Secondary education	74	254	0.9	(0.5, 1.1)	1.8	(0.6, 1.3)	0.607
	College and above	298	858	2.8	(0.3, 0.9)	0.6	(0.4, 1.1)	0.122
Household food insecurity	Secure (ref)	166	588		1		1	
	Insecure	299	900	3.8	(0.6, 1.1)^b^	1.8	(0.6, 0.8)*	0.015
Adolescent food insecurity	Secure (ref)	44	178		1		1	
	Insecure	421	1310	1.7	(0.5, 1.2)^a^	2.6	(0.4, 0.9)	0.132
Dietary diversity score.	Low (ref)	165	614		1		1	
	Medium	77	256	0.8	(0.9, 1.6)^b^	1.2	(0.9, 1.5)	0.535
	High	223	618	0.6	(0.7, 1.5)	1.3	(1.4, 1.6)	0.265
High work load of adolescents	No (ref)	87	344		1		1	
	Yes	378	1144	2.7	(0.6, 0.9)^a^	2.6	(1.2, 3.1)***	0.0001
Cooking place	Sleeping room (ref)	301	1049		1		1	
	Kitchen connected to living room	69	186	2.7	(0.7, 1.4)	1.07	(0.7, 1.6)	0.638
	Separate place	95	253	0.9	(0.6, 1.9)^a^	0.8	(0.5, 1.1)	0.226

* Significant results 1-reference category. Statistical significant (*p < 0.05), highly significant (*** p ≤ 0.0001, ** p ≤ 0.001), * p ≤ 0.05^b^, p ≤ 0.25^a^

During adjusted binary logistic regression analysis, adolescents who had high workloads were 2.6 times [AOR = 2.6; 95% CI 1.2, 3.1] more likely to be associated with thinness compared with those adolescents who didn’t had high workloads. Household food insecurity those insecure households had 80% [AOR = 1.8; 95% CI 0.6, 0.8] higher odds of being thinness compared with those household which secured food. Rural adolescents had 60% [AOR = 1.6; 95% CI 1.3, 2.2] higher odds of being thinness compared with those adolescents who resided in urban areas (Table 3).

### Discussion

The prevalence of stunting was 26.1%. This finding is higher than other studies conducted in Nigeria 17.4% [23], Kenya 16.64% [24]. Discrepancy of proportions might be due to the difference in cultural, feeding habits, and environmental factors of adolescents. This may also due to economical difference among countries [25].

The magnitude of underweight (thinness) was 25.3%. This finding is higher as compared with the other studies conducted in Southeast Ethiopia in 2015 13.6% [26], in Kenya 21.9% [24], and in east central Uganda [27]. The reason for the difference in the prevalence may attribute from the difference in study setting in socio-demographic, cultural and economic characteristics [27]. The other possible justification may result from the current focus of the Ethiopian policy towards nutrition [26].

Stunting was higher among female adolescents (23.8%) than male (21.9%), while thinness was higher among males (27.5%) than female (25.3%) adolescents. This finding is in line with other study conducted in West Hararge, Ethiopia in, 2015 [11] when compared between boys and girls the prevalence of underweight was higher among boys than girls (32.4% VS 10.4%). This might be due to variation of maturation time in boys and girls, for which girls reached maturation earlier than boys [11].

Adolescent stunting was significantly associated with rural residence. Being rural adolescents were more likely stunted than those urban adolescents. This finding is similar to the other studies done from India [28], in Burkina Faso [29], Nigeria [23]. Possible reason might be the fact that adolescents in the urban setting have better access for food, nutrition information and had more educated families.

Stunting is significantly associated with sex of adolescent. Female adolescents were approximately twice more likely stunted than their counterparts of adolescents. Similarly other study in India found that under nutrition was significantly prevalent in girls than boys [28]. Possible reason could be attributed to socio cultural influence at the early age parents give priority care for boys than girls.

Household food insecurity is significantly associated with underweight (thinness) of adolescents. This finding is in line with the other studies conducted in Bangladesh [30], and in Kenya [24]. Possible reason might be due to seasonal variations within countries may affect under-nutrition, particularly stunting and underweight in Ethiopia the prior survey agreed with the long rainy season, when there is generally a lack of food at home [31].

### Conclusion

In Jimma zone, southern Ethiopia significant proportion of underweight and stunting among adolescents and dietary diversity is an independent predictor. There were also identified factors associated with stunting and underweight. Thus, programmers should support adequate nutrition for adolescents.

## Limitations

This study was used cross-sectional study design which can’t determine causality that means temporal sequence between exposure and disease can’t be established.

## Data Availability

All the data that support the findings of this study is available from the corresponding author upon reasonable request in the form of Stata Version 14.

## Abbreviations

AOR: adjusted odd ratio
OR: odd ratio
DDS: Dietary Diversity Score
FAO: Food and Agriculture Organization of the United Nations
FFQ: Food Frequency Questionnaire
FSNP: Food Security and Nutrition Policy
GoE: Government of Ethiopia
IDDS: Individual Dietary Diversity Score
JLFSY: Jimma Longitudinal Family Survey Youth
EDHS: Ethiopia Demographic and Health Survey
VIF: variance inflation factor
